# Neurophysiology of epidurally evoked spinal cord reflexes in clinically motor-complete posttraumatic spinal cord injury

**DOI:** 10.1007/s00221-021-06153-1

**Published:** 2021-07-02

**Authors:** Jose Luis Vargas Luna, Justin Brown, Matthias J. Krenn, Barry McKay, Winfried Mayr, John C. Rothwell, Milan R. Dimitrijevic

**Affiliations:** 1 Center of Medical Physics and Biomedical Engineering, Medical University of Vienna, Währinger Gürtel 18-20/4L, 1090 Vienna, Austria; 2 Department of Neurosurgery, Massachusetts General Hospital, Harvard Medical School, 55 Fruit St, Boston, MA 02114 USA; 3 Department of Neurobiology and Anatomical Sciences, University of Mississippi Medical Center, 2500 North State Street, Jackson, MS 39216 USA; 4 Center for Neuroscience and Neurological Recovery, Methodist Rehabilitation Center, 1350 East Woodrow Wilson, Jackson, MS 39216 USA; 5 Hulse S.C.I. Research Lab, Shepherd Center, 2020 Peachtree Rd NW, Atlanta, GA 30309 USA; 6 Institute of Neurology, University College London, Queen Square, London, WC1N 3BG UK; 7 Department of Rehabilitation and Physical Medicine, Baylor College of Medicine, 1 Baylor Plaza, Houston, TX 77030 USA; 8 Foundation for Movement Recovery, Bolette Brygge 1, 0252 Oslo, Norway

**Keywords:** Spinal cord injury, Epidural spinal cord stimulation, Monosynaptic reflexes, Polysynaptic reflexes, Sustain stimulation, Neural processing

## Abstract

Increased use of epidural Spinal Cord Stimulation (eSCS) for the rehabilitation of spinal cord injury (SCI) has highlighted the need for a greater understanding of the properties of reflex circuits in the isolated spinal cord, particularly in response to repetitive stimulation. Here, we investigate the frequency-dependence of modulation of short- and long-latency EMG responses of lower limb muscles in patients with SCI at rest. Single stimuli could evoke short-latency responses as well as long-latency (likely polysynaptic) responses. The short-latency component was enhanced at low frequencies and declined at higher rates. In all muscles, the effects of eSCS were more complex if polysynaptic activity was elicited, making the motor output become an active process expressed either as suppression, tonic or rhythmical activity. The polysynaptic activity threshold is not constant and might vary with different stimulation frequencies, which speaks for its temporal dependency. Polysynaptic components can be observed as direct responses, neuromodulation of monosynaptic responses or driving the muscle activity by themselves, depending on the frequency level. We suggest that the presence of polysynaptic activity could be a potential predictor for appropriate stimulation conditions. This work studies the complex behaviour of spinal circuits deprived of voluntary motor control from the brain and in the absence of any other inputs. This is done by describing the monosynaptic responses, polysynaptic activity, and its interaction through its input–output interaction with sustain stimulation that, unlike single stimuli used to study the reflex pathway, can strongly influence the interneuron circuitry and reveal a broader spectrum of connectivity.

**Supplementary Information:**

The online version contains supplementary material available at 10.1007/s00221-021-06153-1.

## Introduction

Electrophysiological studies of spinal reflex mechanisms in intact humans have revealed a wealth of detail about individual pathways and their interactions (Pierrot-Deseilligny and Burke 2005). However, most of them have focused on responses to just single afferent volleys, which activate predominantly mono- and disynaptic connectivity, since it is rare for a single afferent volley to be conducted faithfully through multiple synaptic relays. Theoretically, the use of repetitive stimuli can promote temporal summation and reveal a broader spectrum of connectivity (Eccles et al. 1963). However, in humans with intact descending control of spinal excitability, reflexes habituate rapidly, markedly reducing the advantage of temporal summation (Clair et al. 2011).

The situation is quite different in those who have lost voluntary movement control due to a spinal cord injury (SCI). Previous work in SCI-induced motor-complete paralysis has shown that repetitive stimulation of afferent input can result in intricate spinal motor output patterns that evolve gradually over time. For example, plantar flexor withdrawal reflex or stretch reflex evoked by continuous trains of stimuli of constant strength and frequency (1–2 Hz) tend to show amplitude sensitisation, followed by a plateauing and then by habituation throughout tens to hundreds of stimuli (Dimitrijevic and Nathan 1970, 1973). Such behaviours characterise the processing carried out by the spinal circuitry when presented with highly synchronised, low-frequency input. However, the clinical picture after SCI includes more complex changes in motor control, such as partial paralysis and spasticity, that originate from complex processing of peripheral and central input and lead to muscle activation and movement patterns, purposeful and unintentional alike.

In recent years, there has been a resurgence of interest in using implanted epidural stimulation of the posterior roots of the lumbar spinal cord in the rehabilitation of SCI (Courtine et al. 2009; Angeli et al. 2014; Dimitrijevic et al. 2016; Mayr et al. 2016; Taccola et al. 2018). However, the optimal use of such interventions remains highly dependent on a refined understanding of the intrinsic spinal circuitry’s organisation and connectivity that provides input to spinal interneurons (Edgley 2001). While classical reflex studies helped to characterise reflex pathways and their modulation, they rely on peripheral nerve stimulation, subject to long conduction delays to the spinal cord and possible contamination of reflex responses by antidromic activity in motor fibres. In contrast, implanted epidural stimulating electrodes preferentially activate afferent fibres within the dorsal roots (Rattay et al. 2000). Furthermore, because the conduction pathway to the cord is short, epidural stimulation has minimal temporal dispersion, allowing the use of a wide range of input frequencies.

As epidural stimulation activates afferent fibres in multiple dorsal roots bilaterally, a single stimulation pulse results in (presumably monosynaptic) reflex responses in virtually all lower limbs muscles (Rattay et al. 2000; Minassian et al. 2004). At higher stimulation frequencies, epidural stimulation can induce various motor behaviours, including locomotor-like activity (Dimitrijevic et al. 1998). This modulation is likely a result of interneurons (which are activated transynaptically by epidural stimulation) acting on motoneurons. Thus, we suggest that the activation of a population of interneurons is required for complex motor outputs in response to epidural stimulation. This is suported by the observation of long-latency responses (Sayenko et al. 2014), which can be seen as a measure for interneural engagement.

In this context, we analyse the effects of parameter variation in eSCS on repetitively elicited monosynaptic and polysynaptic lumbosacral reflexes and their interaction. We study short and long-latency responses to low-frequency stimulation and relate them to motor outputs at a higher frequency. It is shown how changing input frequency and amplitude can elicit various behaviours in monosynaptic and polysynaptic responses. In particular, it is presented how low-frequency eSCS elicits monosynaptic and polysynaptic spinal reflexes, and how increasing frequency promotes inhibition of monosynaptic responses and facilitates polysynaptic expression. The work presented here provides direct electrophysiological evidence of some basic rules that govern polysynaptic processing.

## Methods

### Patients

Ten individuals with clinically motor-complete S.C.I. (two female, eight male) were recruited (Table 1). The age ranged from 18 to 37 (26.7 ± 5.9; mean ± SD) years, level of injury between C4 and T7, and the time since injury between 2 and 14 years (4.9 ± 3.5 years). The study was approved by the local ethics committee, was conducted in compliance with the Declaration of Helsinki, and all participants gave their informed consent before the procedures.

**Table 1:** Demographic, neurological and neurophysiological data

SID	Sex	Age	Years post-injury	Vertebral level of fracture	AIS	Neurol. level of injury
1	M	22	5	C5-C6	A	C6
2	M	26	4	C4-C5	A	C4
3	M	18	3	C4-C5	A	C4
4	M	25	2	C5-C6	B	C7
5	M	21	3	T7-T8	A	T7
6	M	37	5	C6-C7	A	C6
7	F	25	4	T7	A	T5
8	F	33	3	T5-T6, T10	A	T5
9	M	28	8	C6-C7	B	C6
10	M	33	14	T5-T7	B	T5

### Spinal cord stimulation

Epidural Spinal Cord Stimulation was applied with cylindrical quadripolar electrodes at the tip of a catheter type electrode lead (3487A, Medtronic, Minneapolis, USA) placed in the posterior epidural space centred over vertebral levels from T11 to L1. The four contacts (3 mm each) are distributed along 30 mm and labelled as 0 (most rostral), 1, 2 and 3 (most caudal). Electrode 0 was situated close to the L2 cord segment. An initial coarse positioning was done with the support of fluoroscopy (AP and lateral X-rays). The final position was determined by observing the monitored muscles’ recruitment order when cathodic stimulation on electrode 0, giving preference to the location that evoked the higher responses in the quadriceps (Dimitrijevic et al. 1980; Murg et al. 2000; Pinter et al. 2000). A more detailed description of the placement procedure can be found in previous reports (Murg et al. 2000; Pinter et al. 2000).

In this work, electrode configuration is limited to 0–3 + and 3–0 + , where “ − ” stands for cathode and “ + ” for anode—electrodes 1 and 2 remained inactive. The stimulation pulses were applied with an external stimulator (model 3625, Medtronic, Minneapolis, U.S.A.) configured to deliver monophasic charge-balanced voltage-controlled pulses of 210 μs pulse width.

Stimulation was applied continuously, with a systematic variation of amplitude and frequency. Stepwise changes in amplitude were performed in increments of 1 V and kept unchanged for 8 s. Similarly, the rate was increased between 2 and 100 Hz, again within an interval of 8 s.

In the first part of the assessment protocol, a continuous low-frequency train was applied at the minimum rate of 2 Hz. The intensity sweep started at 1 V and increased with 1 V steps. The intensity was increased until the responses saturated in their peak-to-peak amplitude, the subject perceived any sign of discomfort or the maximum stimulus amplitude of 10 V was reached.

In the second part of the assessment, the intensity sweep was repeated at different frequencies. It started at 2 Hz or 5 Hz and increased until 100 Hz was reached, or the parameter combination caused signs of discomfort to the subject.

### Response monitoring

The response of the nervous system to the eSCS was indirectly analysed via the neuromuscular activity, which was monitored via surface electromyography (sEMG) from the principal lower limb muscle groups—quadriceps (Q), hamstrings (H), tibialis anterior (TA) and triceps surae (TS). An additional channel was used for stimulus artefact recording via electrodes in the lower trunk area, close to the implanted electrodes’ site. This channel was necessary to gain an independent reference trigger, as stimulus artefacts are not visible in the response recordings directly. The sEMG was amplified via a Grass 12A5 system (Grass, Quincy, MA, USA), adjusted to a gain of 5000 and a bandwidth of 50–800 Hz, and digitised at 2 kHz with a 12-bit resolution by a CODAS ADC system (DATAQ Instruments, Akron, OH, USA).

### Data analysis

All data were post-processed in MATLAB (The MathWorks Inc., Natick, MA, USA).

#### Short-latency responses

##### Response amplitude

The stimulation onset for all responses was identified from the artefact recording at the subjects’ lower trunk. The sEMG responses to stimulation pulses were primarily quantified as peak-to-peak voltage (Vpp) of the response within a visually identified window—7–40 ms post-stimulation for Q and H muscles, and 12–45 ms for TA and TS muscles. The quantification with peak-to-peak voltage differs from the area-under-the-curve used for long-latency responses because background (probably polysynaptic) activity could appear during the selected windows. For the purpose of this study, the responses were only considered valid if the Vpp was higher than 30 µV. The responses were also analysed in detail in their morphology in time and amplitude.

The reflexes were verified as reflexes by a double stimuli paradigm (Minassian et al. 2004), which compares the ratio of the first and second response for a 32 ms inter-stimuli interval at the maximum comfortable intensity for each subject.

##### Recruitment curve

For each electrode configuration and muscle, the recruitment curve was estimated to describe the relationship between the short-latency response amplitude (*R*) and the stimulation intensity (*s*). Only the data from stimulation at 2 Hz was used for the recruitment curve since, at these low frequencies, the responses are expected to be stable. However, some fluctuation might appear on the first responses due to post-activation depression mechanisms (Clair et al. 2011). Therefore, to avoid this effect, the first three samples of each combination were not quantified. The recruitment curve was estimated using a Hill-sigmoid function (Gadagkar and Call 2015) with parameter optimisation by the Levenberg-Marquard nonlinear least-mean-squares algorithm (MATLAB, The MathWorks Inc., Natick, MA, USA). Equation 1 shows the Hill-sigmoid function adapted to represent the recruitment curve of large diameter afferents evoking short-latency responses using three parameters: *R*_inf_, the saturation of the response amplitude at a theoretical infinite stimulation intensity; *S*_50_, the stimulus intensity necessary to elicit a response of 50% the amplitude of R_inf_; and m, the slope parameter (Krenn et al. 2020).

1 $$ R\left( s \right) = \frac{{R_{{\inf }} }}{{1 + \left( {s/S_{{50}} } \right)^{{ - m}} }} = \frac{{R_{{\inf }} s^{m} }}{{S_{{50}}^{m}  + s^{m} }} $$

**Fig. 1:**
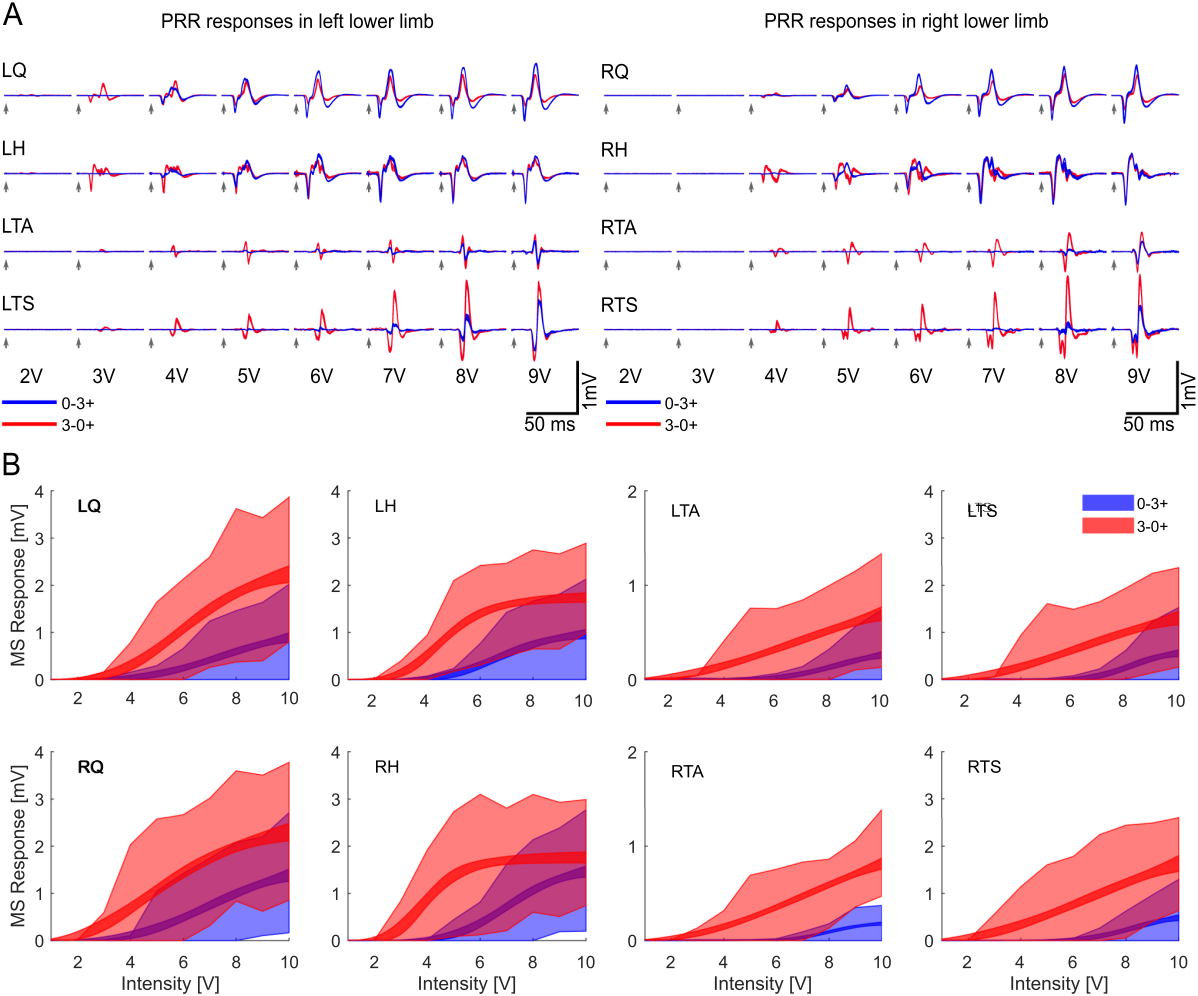
Recruitment differences and effect of voltage field distribution. **A** Monosynaptic responses of the lower limb muscle groups to different intensities and electrode configurations, 0–3 + (blue) and 3–0 + (red). **B** Estimated recruitment curve for nine subjects (SID2 excluded), showing the 95% confidence interval for the mean amplitude (narrowband) and standard deviation of the samples at each intensity (shadowed area) for the lower limbs with both electrode configurations

The parameter estimation was discarded in the following cased: *S*_50_ was equal to the maximum applied intensity, meaning that there is not enough data to estimate the full curve correctly; *R*_inf_ was smaller than 100 µV, which would be physiologically unlikely and would indicate that the maximum intensity was not high enough; or if the signal presented polysynaptic activity on the measured window of short-latency responses—considerable background activity or rhythmic response amplitudes, which are unlikely at 2 Hz stimulation—and caused a drop of the goodness-of-fitness parameter (*R*^2^) below 0.8. For the pooled recruitment curve (Fig. 1B), all fittings were included.

From the recruitment curve estimation, the motor threshold (MT) was determined as the intersection of the tangent of the curve, when *s* = *S*_50_, with the abscissa (Devanne et al. 1997). If the parameter estimation was discarded, the motor threshold was estimated as the minimum intensity that produced a response higher than 30 µV.

#### Long-latency responses

Long-Latency responses were quite variable, appearing in discharges with different latencies, duration, and amplitudes. In the sEMG, these responses are observed as activity above the noise level (> 10 µV) with different durations. In order to discriminate between polysynaptic activity and stochastic motor unit potentials or noise, only discharges of at least 10 ms were quantified as such. Moreover, if two contiguous samples above the noise level are more than 5 ms apart, then those samples were considered part of two different discharges. Finally, to avoid the detection of signal stabilisation after the high-amplitude short-latency response and avoid any influence of the stimulation artefact of the next pulse, the signal was only analysed for long-latency responses on the period from 70 to 400 ms after each stimulus onset.

The quantification of the long-latency responses is based on the area under the curve of the detected discharges, measured in µV s; This option was chosen since it reflects a combination of amplitude and duration of the response, unlike the peak-to-peak or rms voltage, where only the largest samples are considered or the silent periods bias the result. The response amplitude for each pulse is defined as the sum of the area of all the discharges detected.

Unlike the monosynaptic responses, the long-latency responses do not necessarily appear after the first stimuli at threshold intensity, but it might take several repetitions at a minimum stimulation rate. For this reason, the threshold intensity estimated for this type of responses was defined as the one that produced an average long-latency response of 0.1 µV s among the pulses applied at the minimum stimulation rate (2 Hz).

A pooled recruitment curve was estimated with the same procedure as for short-latency responses, calculating the 95% confidence interval for the mean polysynaptic response at each intensity and electrode configuration.

#### Response quantification at different frequencies

The previous two subsections described the quantification methods used at the lowest frequency applied (2 Hz), which has a relatively long pause between pulses, and latency-based discrimination of the type of response is possible. A third quantification method is done to describe the effect of the different stimulation frequency on the overall muscle activity. The peak-to-peak voltage is not a suitable method since it might bias the calculation to consider only the larger single event, e.g. synchronised monosynaptic responses. On the other hand, the area-under-the-curve might be biased toward the lower frequencies, which have a longer period and more activity is taken into account. Therefore, the average root-mean-square voltage (Vrms) was employed to quantify the average response between stimulation pulses at different frequencies. This is preferable since it considers the average activity over the measured time. To avoid quantifying residuals of the stimulus artefact, only the data from 2.5 ms after the stimulus onset until 0.5 ms prior to the next stimulus is quantified.

#### Statistical analysis

Analysis of Variance (ANOVA) was employed to compare the effect of the main factors—subject, electrode configuration, intensity, side, and muscle—on the amplitude of both muscle responses, short- and long-latency. The normality assumption was proof with the Kolmogorov–Smirnov test, which was applied individually to every combination of parameters, given that the mean of the responses was higher than 30 µV. The test shows that less than 6% of the datasets deviate from a normal distribution. The muscle responses were studied as amplitude (peak-to-peak or area-under-the-curve), threshold intensity or maximum response (within the stimulation range applied).

For the analysis, the intensity was treated as a continuous covariant. In addition to the muscle group itself, alternative categories were applied to the group depending on the side (Left/Right) or, alternatively, by its proximal (Q and H) or distal (TA and TS) position. A significance level of *α* = 0.001 was considered for all the statistical tests presented.

## Results

### Monosynaptic responses

The epidural spinal cord stimulation is able to elicit monosynaptic responses bilaterally in all the monitored lower-limb muscle groups (Fig. 1A). A double stimuli paradigm verified the reflex nature of these short-latency responses. The test showed that, on average, the second response was 16 ± 20% the size of the first response, which fits within the expected ratio at the high intensities used (8 ± 1.25 V) and provides evidence for the reflex nature of the responses (Minassian et al. 2004).

The amplitude of the short-latency responses was analysed to evaluate the influence of the electrode configuration, intensity, side, and variability between different subjects and muscle groups. As expected, the data show that the stimulation amplitude [*F* (1, 46,624) = 11,368.65, *p* < 0.0001], the electrode configuration [*F* (1, 46,624) = 3663.73, *p* < 0.0001], intersubject variability [*F* (9, 46,624) = 1677.12, *p* < 0.0001] and muscle group [*F* (3, 46,624) = 709.35, *p* < 0.0001] were all statistically significant. Although close to statistical significance, the muscle side has no significant effect [*F* (1, 46,624) = 7.97, *p* = 0.0048]. This observation suggests that there can be some asymmetry observed between the lower limb muscles—quite common in SCI people—or slight differences in the electrode insertion.

The short-latency responses are summarised in recruitment curves based on intensity sweeps at low-frequency stimulation (2 Hz, Fig. 1B). It is possible to observe that these short-latency responses have a consistent onset (Fig. 1A), and their amplitude is directly proportional to the applied electrical field. As described by the ANOVA test, some variability exists between subjects and muscles. For example, the subject depicted in Fig. 1A has lower thresholds on the left side, presumably due to an asymmetry in the subject’s motor pool excitability. In the same subject, alternating the cathode from electrode 0 (L2 segment) to electrode 3 (30 mm below) also inverted the recruitment preference from Q/H to TA/TS muscles. This is likely because the cathode came closer to the spinal roots that innervate the calf muscles. Figure 1B summarise the peak-to-peak amplitude of the response in the 9 participants for different intensities and electrode configurations, represented by the fitted Hill-sigmoid function and the standard deviation of all the observed samples at each stimulation intensity. Data from SID 2 was excluded from the pooled fitting of the recruitment curves since it showed responses from 0.5 V and reach saturation before 6 V, which was atypical for the rest of the subjects.

A 4-way ANOVA was conducted to compare in detail the effect of the electrode configuration, side, muscle group and subject on the motor threshold of the short-latency responses. The strongest effects were the electrode configuration [*F* (1, 140) = 98.02, *p* < 0.0001] and intersubject variability [*F* (9, 140) = 22.26, *p* < 0.0001].. The muscle group [*F* (3, 140) = 2.0, *p* = 0.1172] and side [*F* (1, 140) = 0.17, *p* = 0.6826] fell irrelevant for modifying the motor threshold. If the distal (TA and TS) and proximal (Q and H) position is tested instead of the muscle group, the muscle position comes closer to significance [*F* (1, 142) = 4.57, *p* = 0.0343] but still below the threshold. This is observed in Fig. 1B, where the distal muscles have, on average, higher thresholds than the proximal muscles. Specifically, the average thresholds for Q, H, TA, and TS were 6.0 ± 2.4 V, 6.25 ± 2.4 V, 6.4 ± 2.9 V and 6.3 ± 2.6 V, respectively for a 0–3 + electrode configuration. For a configuration of 3–0 + , the thresholds were 4.1 ± 1.9 V, 3.3 ± 1.6 V, 4.2 ± 2.0 V and 4.5 ± 2.0 V, respectively (See Supplementary Material 1 for individual thresholds).

In a similar way, a 4-way ANOVA was conducted over the maximum response achieved within the stimulation range applied (1–10 V), the electrode position [*F* (1, 140) = 21.14, *p* < 0.001], the intersubject variability [*F* (9, 140) = 13.52, *p* < 0.0001] and the muscle group [*F* (3, 140) = 10.75, *p* < 0.0001] shown a statistically significant influence. The muscle side has no significant influence on the maximum response achieved [*F* (1, 105) = 1.3, *p* = 0.2577] (See Supplementary Material 2 for individual values). Since the muscle side consistently shows no or very low significance, only the results from one side are shown in some figures and pooled together in others.

### Polysynaptic responses

**Fig. 2:**
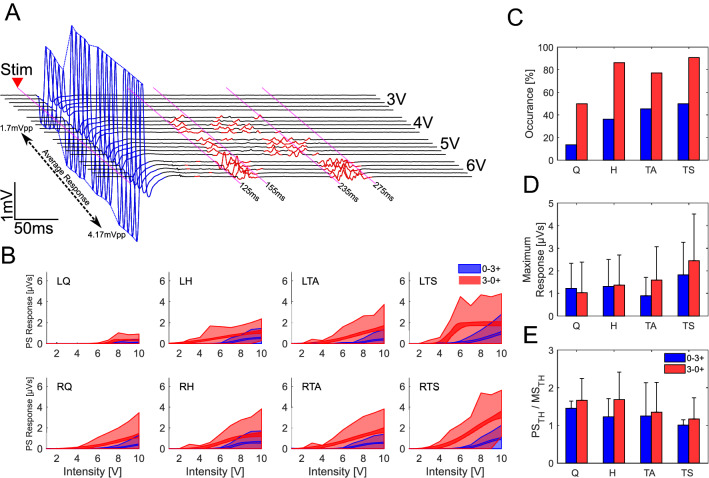
Characteristics of the Polysynaptic responses. **A** Exemplary responses from RH on SID 10 with 3–0 + electrode configuration. The sEMG show in blue the short-latency (monosynaptic) responses, appearing after ~ 7 ms, and, in red, the long-latency (polysynaptic) responses, which appear in two groups with latencies of around 130 ms and 260 ms. Both types of responses evolve when the stimulus intensity is increased. **B** Estimated recruitment curve for eight subjects (SID2 and SID8 excluded), showing the 95% confidence interval for the mean amplitude (narrowband) and standard deviation of the samples at each intensity (shadowed area) for the lower limbs with both electrode configurations. **C** Shows the percentage of polysynaptic activity occurrence on each muscle group (both sides grouped together). **D** shows the mean maximum response for each muscle and electrode configuration (SID8 excluded due to continuous activity not associated with the stimulation). Finally, **E** Shows the relative threshold of the polysynaptic activity compared to the monosynaptic activity

In addition to the monosynaptic responses, there can be long-latency components. These were observed at different latencies and durations across muscles and subjects. Figure 2A has an expanded time scale to illustrate these components for one subject (SID 10). It shows the formation of two groups of polysynaptic discharges when the intensity increased. One group has a latency of 135 ± 4.6 ms and 26 ± 5.6 ms duration, and the other has a latency of 239 ± 2.8 ms and 41 ± 4.5 ms duration. It is important to notice that the grouping of polysynaptic responses was not homogeneous across the subjects and, as observed in the first column of Fig. 4B, D, multiple or single polysynaptic activity groups can be elicited.

The recruitment curves show a less typical behaviour, with higher thresholds and spikes in the standard deviation (Fig. 2B). A 4-way ANOVA was conducted to evaluate the influence of the electrode configuration, intensity, subject and muscle in the long-latency response amplitude. The results show that Intensity [*F* (1, 46,625) = 5342.13, *p* < 0.0001], electrode configuration [*F* (1, 46,625) = 2874.97, *p* < 0.0001], intersubject variability [*F* (9, 46,625) = 866.39, *p* < 0.0001] and muscle [*F* (3, 46,625) = 810.26, *p* < 0.0001] all have a significant effect on the long-latency response amplitude. Similar to the monosynaptic responses, the polysynaptic activity was elicited easier with the 3–0 + configuration (Fig. 2C). A summary of the maximum responses is also shown in Fig. 2D.

In those signals that showed polysynaptic responses, the effect of electrode configuration, intensity, subject and muscle on the number of polysynaptic discharges was analysed. Similar to the response amplitude, the muscle [*F* (3, 11,235) = 230.51, *p* < 0.0001], intensity [*F* (1, 11,235) = 217.96, *p* < 0.0001], intersubject variability [*F* (9, 11,235) = 175.65, *p* < 0.0001] and electrode configuration [*F* (1, 11,235) = 83.27, *p* < 0.0001] all have a significant effect on the number of polysynaptic discharges. Although all *p* values were highly significant, it was possible to identify the subject and the muscle group as the first and second most relevant variables. It is important to notice that, unlike short-latency responses where stimulation intensity only increased the response size, on long-latency responses, the intensity promotes the polysynaptic activity synchronisation, as observed in Fig. 2A, where the activity is grouped in 2 discharges of shorter duration but higher amplitude.

In Fig. 2A, the threshold for evoking long-latency components is 4 V, in contrast to the 2.3 V (as estimated by the Hill-sigmoid function) required to evoke the short-latency responses. Interestingly, the polysynaptic responses do not appear after the first stimulus at threshold intensity, but they slowly appear after a few stimulation pulses (Fig. 2A). This suggests a time and rhythmic dependency of the interneural circuits and a direct way to modify the motor output.

The average thresholds for Q, H, TA, and TS were 8.3 ± 1.5 V, 7.6 ± 1.3 V, 8.1 ± 1.2 V and 7.8 ± 1.4 V, respectively for a 0–3 + electrode configuration. For a configuration of 3–0 + , the thresholds were 7.0 ± 1.8 V, 6.0 ± 2.0 V, 5.9 ± 1.8 V and 5.4 ± 1.9 V, respectively (See Supplementary Material 3 for individual thresholds). These values exclude SID2, whos data bias the result to look similar between both electrode configurations. While the threshold to elicit polysynaptic activity was usually higher (Fig. 2E), there were few exceptions. A 3-way ANOVA was performed to analyse the effect of the intersubject variability, electrode configuration and muscle group in the polysynaptic threshold. It is observed that the electrode position [*F* (1, 85) = 87.95, *p* < 0.0001] and intersubject variability [*F* (9, 85) = 30.8, *p* < 0.0001] had a significant effect on the threshold for this kind of responses. Although close to our significance level, the muscle group does not significantly affect the threshold [*F* (3, 85) = 4.62, *p* = 0.0048].

Unlike monosynaptic responses, polysynaptic responses do not necessarily appear in all subjects, but their occurrence varies. The highest occurrence was achieved with the electrode configuration 3–0 + with a preference for the flexor muscles Fig. 2C. Interestingly, polysynaptic responses can also synchronise between muscles and exhibit responses with the same or opposite phase (not shown). A similar effect has been previously reported (Hofstoetter et al. 2015). This synchronisation shows the complexity or the interneural circuitry that interact among different neuron pools.

### Interaction between monosynaptic and polysynaptic responses

**Fig. 3:**
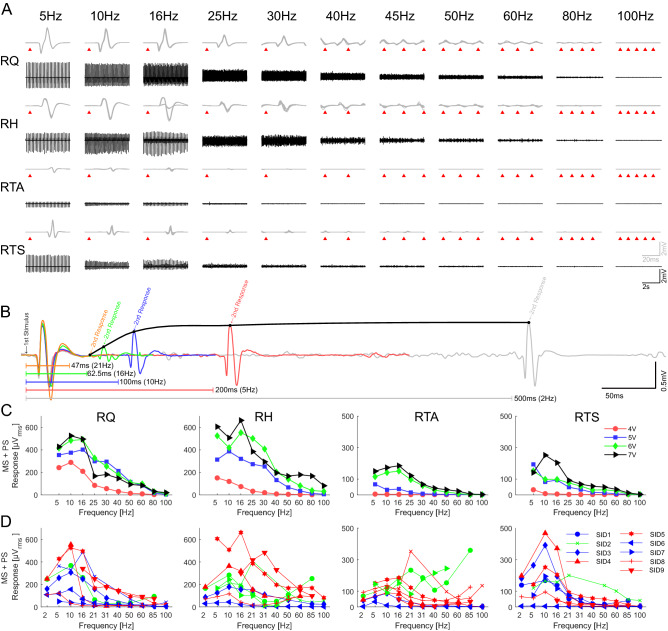
Modulation of neuromuscular responses due to frequency changes. **A** Neuromuscular responses of right lower limb muscles to stimulation frequencies from 5 to 80 Hz at 5 V (SID 5). Upper traces show a detail of 10 superimposed responses (grey) with a mark (red triangle) on the stimulation times. The lower part (black) shows the activity in the range of 1–6 s (0–3 +). **B** shows the overlap of the first two responses from SID 3 when 7 V was applied at different frequencies (2, 5, 10, 16 & 21 Hz) in RH (periods between stimulus are marked with the colour lines); the black line marks the maximum value of the second response at each frequency. **C** Summary of the decay on EMG activity (mean rms) of the right lower limb muscles when the frequency increased from 5 to 100 Hz at fixed intensities (SID 5, 0–3 +). **D** Summary of the EMG activity on the right limb muscles from 9 subjects when the frequency increased from 5 to 100 Hz at the maximum intensity applied on each subject. In blue, green, and red are the cases where the motor output at 85 Hz stimulation at highest frequency are suppressed, tonic or rhythmic, respectively (see Table 2)

An increase of the stimulation frequency at low intensities produced the habituation of monosynaptic responses on all subjects. Figure 3A shows the effect of different stimulation frequencies on the short-latency responses (10 superimposed responses) as well as a 5 s segment in a single individual (SID 5) with 5 V pulses. As the frequency increases, particularly above 16 Hz, the responses decrease and almost disappear at the high-end frequencies.

Figure 3B illustrates another example of this trend on a different individual (SID 3). Here, the first responses to trains of stimuli at 2, 5, 10, 16 and 21 Hz are superimposed at the start of the trace. The second, later, response comes at different times depending on the frequency of stimulation. In this individual, the short-latency response to the second stimulus of the train is absent at 21 Hz, and it is much smaller at 16 and 10 Hz, illustrating how quickly the short-latency response habituates.

It is essential to notice that, although the monosynaptic responses tend to decline with higher frequencies, this is not a linear process, and fluctuation on the size of consecutive responses is possible. An example of this is shown with 16 Hz stimulation in the RH of SID5 (Fig. 3A), where the monosynaptic responses consistently alternate between 2 different amplitudes.

**Fig. 4:**
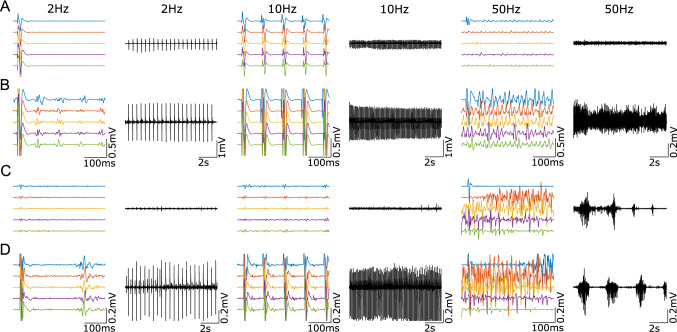
Behavioural differences on frequency effects when stimulation intensity elicits polysynaptic activity. **A** Show the RH from SID 1 at 5 V when 2, 10 and 50 Hz are applied. **B** Shows the same but at stimulation intensity of 7 V, which could elicit polysynaptic responses at 2 Hz. **C** Show the RTA from SID 4 at 6 V when 2, 10 and 50 Hz are applied. **D** Shows the same but at stimulation intensity of 8 V, which could elicit polysynaptic responses at 2 Hz

In Fig. 3C, the mean average of the responses—root mean square voltage—in one subject (SID 5) are shown for different intensities. It is observed that the decreasing trend observed in Fig. 3A is not absolute and that changing the frequency might also lead to the fluctuation in the motor output, depending on the stimulation intensity. In this case, the ultimate effect at 100 Hz is the complete suppression of activity in all muscles but in the hamstrings, where rhythmic activity can be observed (not shown). The suppression trend deviates, especially when the intensity is above the polysynaptic activity threshold, which might change the behaviour once it appears (Fig. 4).

Figure 4A, B show data from the RH of a single individual at 2, 10 and 50 Hz using stimuli subthreshold (A) or suprathreshold (B) for evoking longer-latency responses. Figure 4C and D are similar but from the RTA muscle of a different individual. In Fig. 4B, the longer-latency responses visible at 2 Hz are no longer present at 10 Hz, presumably because motoneurons are refractory following the large monosynaptic response that follows each stimulation pulse. At 50 Hz, the short-latency response has disappeared due to habituation (also in Fig. 4A), and low amplitude continuous EMG activity can be seen. In Fig. 4D, long-latency responses are present at 2 Hz and 10 Hz with partial occlusion of the components with a longer latency than the inter-stimulus interval. At 50 Hz, SID 4 showed rhythmic activity at both intensities, subthreshold (Fig. 4C) and suprathreshold (Fig. 4D). However, rhythmical activity is only sustained when suprathreshold stimulation is applied (Fig. 4D), while for lower intensities, such activity attenuated after a few seconds (Fig. 4C). Interestingly, the amplitude of the rhythmic activity in Fig. 4C is larger than those of short- and long-latency responses at lower frequencies. These observations suggest that at least one additional variable is necessary to elicit rhythmical activity—the central state of excitability [ref]—and methods to quantify it are still to be developed.

**Fig. 5:**
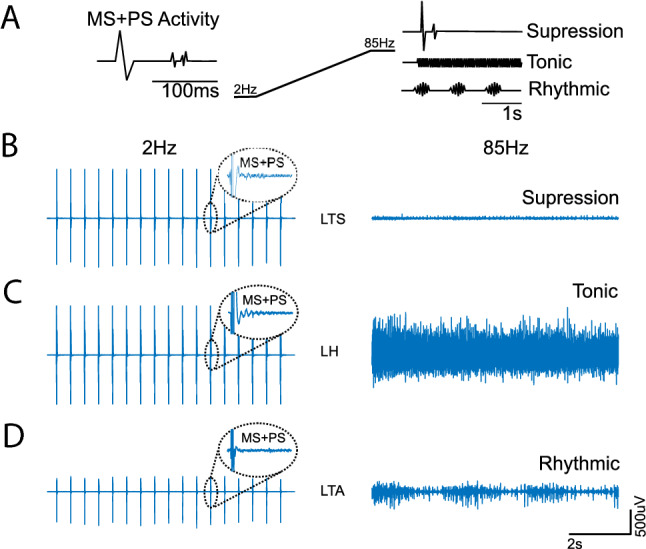
Different behaviours elicited by sustain eSCS. **A** Exemplifies how, when monosynaptic (MS) and polysynaptic (PS) activity is elicited, the motor output at high frequencies can turn tonic, rhythmic or suppressed. For example, when MS + PS activity is elicited in SID 1, **B** LTS is suppressed when 85 Hz stimulation is applied. Simultaneously, **C** LH becomes tonic, and **D** LTA becomes rhythmic

Although suppression at high frequencies is possible regardless of the intensity, under polysynaptic supra-threshold stimulation, the muscle activity might also turn tonic or rhythmical (Fig. 5 and Table 2). It is important to notice that these alternative behaviours are sometimes represented with the increase of the average root mean square voltage of the responses (Fig. 3D), although clear patterns can be observed even when the estimated response amplitude is small (Fig. 3D).

**Table 2:** Motor output observed on each muscle at maximum intensity during 85 Hz (or closest frequency applied) stimulation

SID	Electrode configuration	Frequency	Intensity	LQ	LH	LTA	LTS	RQ	RH	RTA	RTS
1	3–0 +	85 Hz	5 V	S*	T*	R*	S*	T*	T*	T*	S*
	0–3 +	85 Hz	7 V	S*	T*	S*	S*	T*	T*	T*	S*
2	3–0 +	80 Hz	10 V	R*	T*	R*	T*	S*	T*	R*	T*
	0–3 +	80 Hz	10 V	T*	T*	S*	S*	T*	T*	R*	S*
3	3–0 +	85 Hz	8 V	S*	R*	S*	S*	S*	S*	S*	S*
4	3–0 +	60 Hz	8 V	R*	R*	R*	R*	R*	R*	R*	R*
	0–3 +	60 Hz	8 V	R*	R	S	S	R*	R	S*	S*
5	0–3 +	80 Hz	7 V	R*	T*	R*	R*	R*	R*	R*	R*
6	0–3 +	85 Hz	10 V	S	S	S	S	S	S	S	S
7	3–0 +	50 Hz	9 V	S*	T	S	S	S	T	T*	S
	0–3 +	50 Hz	10 V	R*	R	R*	R*	R*	R	R*	R*
8	3–0 +	85 Hz	8 V	R*	R*	R*	R*	R*	R*	R*	R*
9	3–0 +	85 Hz	7 V	R^NA^	R^NA^	R^NA^	R^NA^	R^NA^	R^NA^	R^NA^	R^NA^

*S* suppression (mean Vrms < 40 uVrms), *T* tonic (Vrms ≥ 40 uVrms), *R* rhythmic (if a rhythmic activity is visually confirmed regardless of the mean Vrms)

*Indicates that some level of PS responses was observed at the lowest frequency (< 10 Hz). NA Information of PS responses not available due to initial frequency ≥ 10 Hz

## Discussion

### General overview of the data

In the data presented, we have used bipolar epidural electrical stimulation of afferent nerve fibres in the posterior roots to probe alteration of excitability and interneuron processing after SCI. With an appropriate configuration, stimulation pulses could evoke short-latency, short-duration responses (probably mono- and oligosynaptic) as well as long-latency, long-duration responses (probably polysynaptic) in all main lower extremity muscle groups (Fig. 2). The threshold was generally lower for the short-latency monosynaptic response component, consistent with the selective activation of the largest diameter afferent group I fibres. With further increase of intensity, a pronounced second threshold led to additional later responses, suggesting the co-activation of smaller diameter group II fibres. The monosynaptic responses increase in amplitude with stimulus intensity, indicating that the monosynaptic activation of the motor pool increases until it reached saturation, when all associated motor units react simultaneously to the central (Fig. 1); however, they habituate rapidly at higher stimulation frequencies, above ~ 15–20 Hz (Fig. 3). This confirms that the reactivation of the same motor pool by the same input is prevented by the post-activation depression of the monosynaptic reflex (Hultborn et al. 1996)**.**

Asynchronous polysynaptic responses are seen in all muscles, but their occurrence, onset delay and duration vary between individuals, muscles and stimulation setup (Fig. 2). They are visible at low stimulation frequencies (< 10 Hz), but at higher frequencies (> 50 Hz), when the monosynaptic response has habituated, they are often clearer (Fig. 4). In addition to these discrete monosynaptic and polysynaptic responses, stimulation, particularly at high intensities, could produce prolonged periods of sustained EMG activity (“spasms-like”). It probably indicates that the synchronous activation of whole motor pools has the disadvantage to not only prevent the reactivation of the same motor pool by the same input (monosynaptic pathways) but also by other inputs (polysynaptic pathways), as is suggested by the “incompressible” silent period following the early EMG response (Ashby 1995).

In sum, single electrical stimuli provide a tool for the primary assessment of connectivity and excitability of spinal motor neurons, with limitation to immediate responses by simultaneously recruited neurons. However, functional assessment of more complex interneural networks in the spinal cord requires different methodological strategies. We show that the spinal polysynaptic processing can be investigated when (1) the stimulation intensity range is sufficient to induce both short- (monosynaptic) and long-latency (polysynaptic) responses, and when (2) the stimulation frequency range is sufficient to induce both suppression of short monosynaptic responses and facilitation of interaction of long-latency responses. Therefore, we suggest that repetitive stimulation is an important additional modality for the assessment of the processing behaviour of networks with multiple (polysynaptic) interconnections.

Within the included subjects, 3/10 are classified as AIS B, indicating a sensory incompleteness. Statistically speaking, when the AIS classification was used instead of the subject, it reduced the significance level compared to the intersubject variability influence. Although the AIS classification remains significant in most cases, the *p*-value was higher than our significant level in the case of maximum short-latency response and threshold intensity for both types of responses. This could mean that the influence of the AIS scale derives from the influence of the intersubject variability. Physiologically, it might be explained because the sensory signal reaches the interneuron network in both cases, so the influence of those inputs is integrated in both, AIS A and AIS B. The only difference is that for sensory incomplete, the signals could reach the brain and be interpreted as sensation. However, being both cases motor-complete, the possible supraspinal influence in response to the sensation is limited.

### Recruitment of “monosynaptic” and polysynaptic pathways

In the data presented here, the posterior root stimulation intensity was below the threshold for direct activation of motor fibres in the anterior root. As expected from previous work (Rattay et al. 2000), stimulation occurred preferentially close to the cathode (Fig. 1). The short-latency response had all the mono and oligosynaptic pathway characteristics: constant latency, short duration, and low threshold (Troni et al. 1996; Rattay et al. 2000). This is further confirmed by the double pulse paradigm that, at 32 ms inter-stimuli interval, shows a second response strongly reduced or entirely suppressed, which is characteristic of mono- and oligosynaptic reflexes (Minassian et al. 2004). We presume it was produced mainly by activation of low threshold, larger diameter primary sensory afferents.

The monosynaptic responses increased in size and eventually saturated at high intensities, compatible with spatial recruitment of motor pools by low threshold afferent fibres due to peripheral nerve stimulation (Pierrot-Deseilligny and Burke 2005). Although the latency of these responses varies, the differences were of a couple of milliseconds, accounting mainly for the distance from the stimulation site and the muscle—thigh muscles have shorter latency than calf muscles. The responses were elicited simultaneously in all muscles, probably due to synchronous activation of their afferent fibres in the posterior roots. The data shows that the stimulation setup—intensity and electrode configuration—can effectively modify the voltage field and steer these responses. For example, the electrode configuration can effectively change the muscles’ activation threshold, showing a general tendency to have lower thresholds for the thigh muscles than those of TA and TS.

In addition to the monosynaptic responses, there can be long-latency components. These responses are likely to have a polysynaptic nature; therefore, they are often characterised by smaller amplitudes and more disperse and unsynchronised activity (Fig. 2A), and longer acquisition time is needed for proper visualisation. As shown in Fig. 2C, these components are not observed in every combination of subject, electrode configuration and muscle, and the occurrence is strongly influenced by the electrode configuration, muscle group and subject. Like the short-latency responses, the long-latency responses are elicited easier when the cathode was at contact 3.

The delayed and longer-lasting polyphasic activity was generally evoked at intensities higher than the monosynaptic response threshold. This difference could mean that such polysynaptic responses require a higher proportion of low threshold fibres to be activated, the recruitment of a different higher threshold population—type II afferents—or a combination of both. It was also observed that the stimulation’s rhythmicity influences the appearance of these responses since, at threshold intensity, the responses do not necessarily appear from the first stimulus, but it took a build-up period for the responses to appear. Like the monosynaptic responses, the intensity and electrode configuration played a major role in steering the response amplitude; however, the intersubject variability and muscle are the major factors in the shape of the response (number of discharges). Since, intersubject variability was only the third most important in monosynaptic responses. This indicates that the expected variability due to methodological procedures (e.g. skin preparation, electrode placement) or superficial anatomical characteristics of the subject (e.g. muscle size, the thickness of skin and fat layers) was kept under control. Therefore, it is possible to assume a highly individualise variation that mainly influences the polysynaptic pathways. Two reported factors with such characteristics are the post-SCI anatomy reconfiguration (Kakulas and Kaelan 2015) and the unique neurophysiological excitability state inherent to each subject, and the temporality of the measurement (Dimitrijevic et al. 2016). It has been shown that these variants, described as the influence of residual descending pathways, constitute a significant component to predict the development of spasticity in SCI people (Sangari et al. 2019) and might explain why in some subjects, no polysynaptic activity was elicited even though strong monosynaptic responses were observed. Besides the inclusion of clinically motor-complete SCI people only, neither the new post-SCI anatomy nor the current excitability state is assessed within our protocol.

The stimulation intensity has an interesting influence on the number of polysynaptic discharges elicited. Due to the quantification method, this could mean that the intensity either evoke continuous polysynaptic activity with fewer interruptions during the analysed time window or that, as mostly observed in the recordings, the intensity facilitates the synchronisation of the fired motor units to form groups. An example of this is shown in Fig. 2A, where a 3 V stimulus recruited a single large synchronous monosynaptic response, but at 4 V and above, there was an additional asynchronous activity that groups at 135–161 and at 239–280 ms when the stimulation rises, similar to the polysynaptic response to a short, high-frequency stimulation train to the tibial nerve (Dietz et al. 2009)**.** These longer-latency stimulus-locked events suggest substantial additional processing of the input by distributed central spinal circuitry.

### Effect of repetitive stimulation

The monosynaptic response to repetitive stimulation was constant or slightly facilitated when applied at frequencies of 2–10 Hz but grew smaller at frequencies above 15 Hz. The degree of facilitation at around 10 Hz varied between individuals (e.g., Fig. 3D), but the depression at low intensities was common to all. Reflex depression occurred as early as the second stimulus of a train (Fig. 3B), suggesting that it follows every single response. At high frequencies of stimulation, this could have been because the second stimulus occurred during the period of motoneuronal hyperpolarisation following the first reflex response. However, this cannot explain the continued response’ depression to the third and subsequent stimuli since the second stimulus did not evoke a response. The implication is that reflex depression occurs in the afferent pathway and motoneuronal activation. Similar behaviour has been observed in many previous animal and human experiments, with the reflex depression, or habituation, due to a combination of presynaptic and postsynaptic mechanisms (Eccles and Rall 1951; Delwaide 1973; Hoehler et al. 1981; Hofstoetter et al. 2015). Another mechanism could be a parallel process activated by alternative polysynaptic pathways unaffected by postsynaptic depression of primary afferents (Lamy et al. 2005) at low intensities, and primary and secondary fibres at higher ones.

Interestingly, the frequency at which reflex depression occurs is far higher than that in the intact cord, where both H-reflexes and posterior root reflexes in individuals at rest show substantial depression at 1 Hz or less. Compared to rest, the frequency-dependent H-reflex depression is substantially reduced during voluntary contraction in individuals with intact nervous systems, resulting in little loss of amplitude even at rates of 4 Hz or more (Clair et al. 2011). Presumably, the increased activity during voluntary contraction changes the properties of synapses in the reflex pathway of a reduced transmitter release from previously activated fibres, which is thought to be the primary source of H-reflex depression (McNulty et al. 2008). A related effect may allow even higher frequencies (up to 10–15 Hz) after spinal transection.

As supported by the data and behaviour observed during the measurement, the polysynaptic activity can be elicited either by very high intensity, or a lower intensity if combined with repetitive stimulation. However, unlike high-intensity stimuli, polysynaptic activity elicited by stimulation at threshold intensity starts to appear only after a few stimuli. Since polysynaptic activity elicited by a single pulse can persist for 500 ms or more (Dimitrijevic and Nathan 1967), it is difficult to determine precisely how they are affected by repetitive stimulation at > 2 Hz since the polysynaptic activity of one stimulus could influence the response to the subsequent stimulus.

The suppression trend of monosynaptic responses is well studied (LLOYD and WILSON 1957; Van Boxtel 1986; Lamy et al. 2005). Lamy et al. showed that although post-activation depression of monosynaptic pathways grows stronger with higher frequencies, activity in alternative (polysynaptic) pathways fed by the group I afferents remains unaffected or even grow (Lamy et al. 2005). Therefore, when the frequency is increased, Ia monosynaptic connections are eventually blocked. However, if the input to the interneurons is adequate, the polysynaptic system can increase the excitability of the αMN, so a few Ia monosynaptic potentials are enough to trigger a detectable muscle response. Then, at higher frequencies (> 50 Hz), the monosynaptic responses are entirely blocked, and the activity of the polysynaptic system could be high enough to drive the αMN on its own. This is observed in Fig. 4, where the monosynaptic component is virtually missing with stimulation at 50 Hz, but if the intensity is raised to recruit polysynaptic activity (Fig. 4B and D), this last one persists even at 50 Hz. The implication is that high-frequency stimulation preferentially engages polysynaptic pathways over monosynaptic responses. Interestingly, low-intensity stimulation elicited polysynaptic activity in some exceptional cases, even if monosynaptic responses were not observed in the muscle, which could be due to temporal facilitation due to descending influence crossing the SCI or excitatory input coming from interneurons activated by different motor pools (propriospinal tracts). This is consistent with the previous observation that the central state of excitability plays a critical role.

In summary, we propose that polysynaptic activity is visible at low stimulation frequencies as a direct response on the motor units. However, in the middle-range frequency, their presence can be observed in the neuromodulation of monosynaptic responses (Minassian et al. 2004; Hofstoetter et al. 2015; Danner et al. 2015) or, at higher frequencies (> 50 Hz), as a patterned activity (tonic or rhythmic) expressed on the muscles (Figs. 4D and 5), where responses cannot be tracked back to any specific stimulus.

**Fig. 6:**
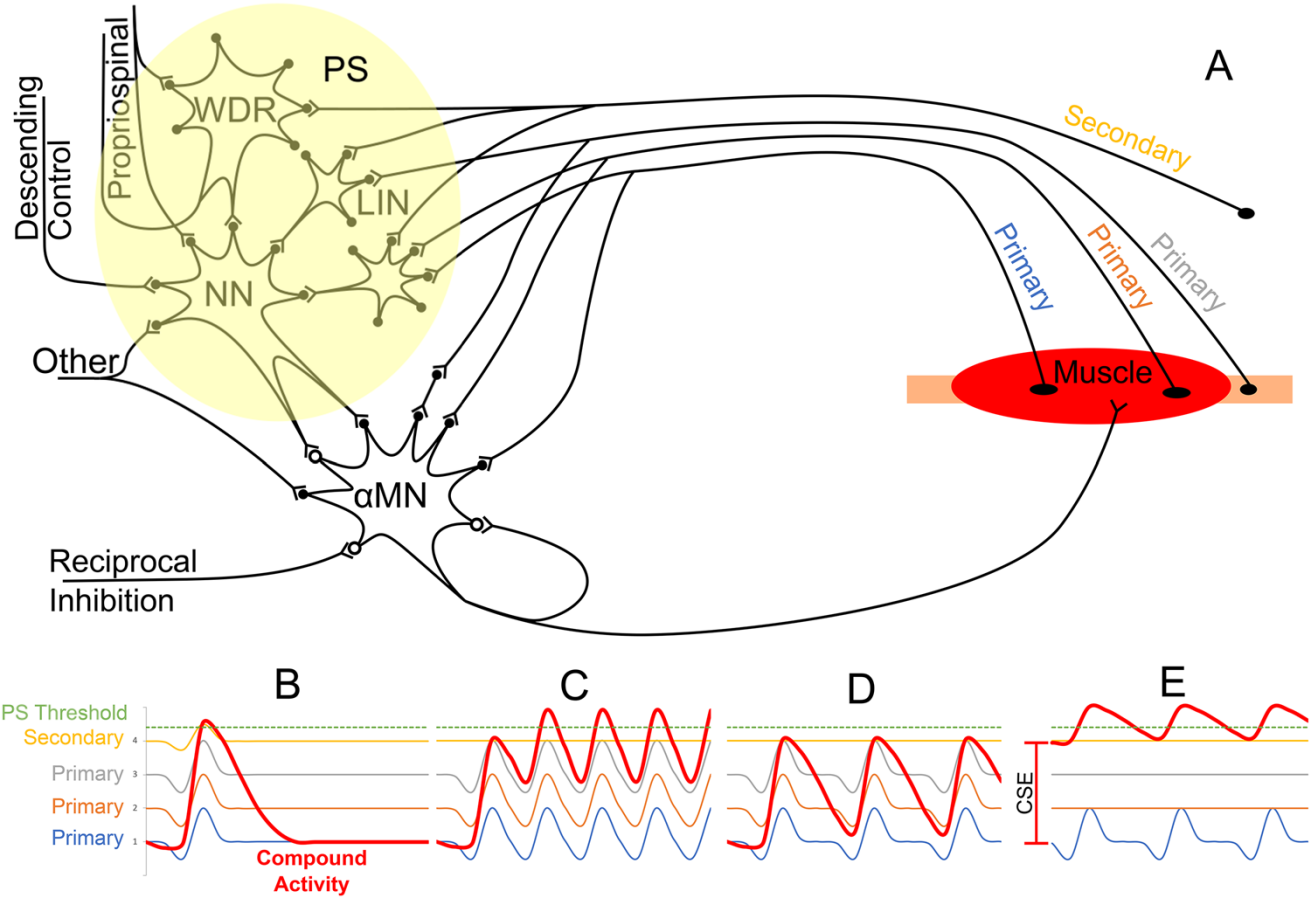
Representation of a possible mechanism behind the activation of the polysynaptic system (PS, composed by *NN* neural network; *WDR* wide dynamic range interneurons; *LIN* local interneurons) based on three elements: primary afferents, secondary afferents, and central state of excitability (CSE). **A** shows the representation of a muscle group with multiple (3×) primary and (1×) secondary afferents, as well as additional inputs conforming the CSE: descending control, propriospinal network, other inputs. The polysynaptic system can be activated by **B** a high-intensity stimulus, which can depolarise primary and secondary fibres to reach the threshold on each stimulus—for this type of stimuli, frequency does not play a role (e.g. 2 Hz stimulation in Fig. 4B and D); **C** it can also be activated by a medium intensity stimulation at high frequency, so a subthreshold potential builds up, and after a few pulses, it reaches the threshold for polysynaptic (e.g. Figure 2A); **D** if the combination of the three inputs—CSE, primary and secondary afferents—is not ideal, the threshold to activate the polysynaptic system is not reach, and the “suppression” trend with increased frequencies is maintained (e.g. Figure 4A); E) If the central state of excitability (CSE) is high, the polysynaptic system can be activated by a low intensity/low-frequency stimulation (e.g. Figure 4C). Once the polysynaptic system is activated, it can neuromodulate the αMN activity to suppress or facilitate it

Figure 6 shows the proposed mechanism to activate the interneuron system (represented as a single neuron here) based on the data presented here, supported by observations across the literature (Edgley 2001; Brownstone and Bui 2010). While primary afferents have mono- and oligo-synaptic connections to the motoneurons, they also branch to a limited area of the polysynaptic system (Brownstone and Bui 2010). On the other hand, higher threshold secondary fibres do not synapse directly with the motoneuron but instead spread across multiple areas of the interneuron circuitry (Brownstone and Bui 2010).

Based on the proposed mechanism, one way to engage the polysynaptic system is by applying high-intensity stimulation. This kind of stimulation would activate all primaries and secondary fibres, producing enough postsynaptic excitatory potentials (PSEP) to trigger the polysynaptic response on the motoneurons (represented as a single motoneuron here) (case Fig. 6B). Alternatively, when the intensity does not produce enough excitatory input to cross the polysynaptic circuitry, combining it with an appropriate repetitive stimulation can compensate and build-up (after some pulses) the necessary input to trigger the polysynaptic responses (case Fig. 6C). This kind of activation is observed in Fig. 2A, where the polysynaptic responses take some time to establish at 4 V. If the combination of intensity and frequency is inadequate, then the polysynaptic threshold will not be reached, and polysynaptic expression will not be observed (case Fig. 6E). Finally, the central state of excitability can modify this behaviour, increasing or decreasing the thresholds so that the activation of a few primary fibres would be enough to engage the polysynaptic circuitry (case Fig. 6D). This is observed in Fig. 4C, where low-intensity stimulation triggered rhythmic activity for a few seconds. It is also consistent with observations that the larger the residual influence (surviving axons) across an SCI, the more likely it is to observe spasticity (Sangari et al. 2019), an expression of a high CSE. It is important to notice that polysynaptic activity can be expressed as active suppression, which could be the case for some subjects’ muscles leading to suppression in this study. However, this case cannot be identified in the current setup, but methods to detect this kind of active suppression has been previously reported (Dimitrijevic and Nathan 1971).

Understanding and predicting how the polysynaptic circuitry will be expressed on the muscle activity is still to be elucidated.

### Clinical relevance

The methodology described here allows the characterisation of dynamic underlying mechanisms at a resting state, translating a clinical condition into a neurophysiological model. These human models allow gathering information about functional connectivity in the spinal cord, which is crucial for developing neuromodulation approaches to restore movements after SCI, and it is limited to in vitro studies (Edgley 2001). It also provides the basis to compare it with experimental animal models, where anatomical and physiological alterations are under control, and extrapolate the results based on evidence rather than speculation.

The neurophysiological assessment described in the methodology can complement the clinical classification and, at the same time, provide information to test underlying mechanisms in human. The results explain, for example, why a non-tailored stimulation might lead to an increase of spasticity rather than ameliorating it. The data also shows that, although some frequency ranges are assumed to produce a specific motor output—tonic (< 15 Hz), rhythmic (15–50 Hz) or suppression (> 50 Hz)—in reality, those are guidelines, and the final motor behaviour will depend on the stimulation site, frequency, intensity and, the subject’s central state of excitability.

Thus reported neurophysiological features of constant short and longer latencies spinal reflex responses and their interaction should be part of the protocols when deciding parameters for stimulation site, strength, frequency, interaction of externally controlled repetitive spinal cord stimulation. αMN.

## Conclusion

Consistent with other works, here we show how repetitive stimulation of the lumbar spinal cord can evoke simultaneous reflexes in all monitored lower limb muscles on paraplegic SCI subjects. It is shown how monosynaptic reflexes, evoked by intensities near threshold, progressively decrease until complete absence when the stimulation frequency increases.

Beyond confirming these observations, our analyses show that polysynaptic components are more common to appear at higher intensities; however, their threshold is not constant and might vary with different stimulation frequencies and the individual central state of excitability, which speaks for its temporal dependency. These components could be observed as direct responses, as neuromodulation of monosynaptic responses or driving the muscle activity by themselves, depending on the stimulation frequency level.

An even more important conclusion is that although there is a trend to suppress monosynaptic responses with increasing frequency, this could deviate if polysynaptic activity is triggered. In this case, the motor output becomes an active process expressed as suppression, tonic or rhythmical activity. Interestingly, statistical analyses suggests the presence of a highly individualised variable, often referred to as central state of excitability, which facilitates the activation of the polysynaptic activity more than any of the tested eSCS parameters. This variable is presumably a key to predict the motor output for each individual, and methods to assess it are still to be elucidated.

The present results are a limited description of the complex behaviour of spinal circuits deprived of voluntary motor control from the brain and in the absence of any other inputs. However, the distribution of excitability in these circuits, and hence the functional outcome, is likely to change in the presence of conditioning stimulation. Future work will examine the influence of limited descending input in participants with discomplete and incomplete lesions, as well as the effect of sensory inputs from other parts of the body (Dimitrijevic 1987). Finally, it should be noted that we have only examined the effects of sustain stimulation; the effects of stochastic stimulation can be different (Dimitrijevic et al. 1972).

## Supplementary Information

Below is the link to the electronic supplementary material.

Supplementary file1 (DOCX 31 KB)

## Data Availability

The datasets generated during and analysed during the current study are not publicly available since additional analysis is undergoing.
