# Supplementary material for: Neurophysiology of epidurally evoked spinal cord reflexes in clinically motor-complete posttraumatic spinal cord injury
Source: Exp Brain Res. 2021 Jul 2;239(8):2605–20. doi: 10.1007/s00221-021-06153-1 (PMC8354937; doi:10.1007/s00221-021-06153-1)
Supplement: Supplementary file 1 — Supplementary file1 (DOCX 31 KB) [file 221_2021_6153_MOESM1_ESM.docx]

| Supplementary Material 1. Summary of Monosynaptic Threshold [V] for each subject, electrode position (EP) and muscle. NA indicates that no reflexes were elicited on that muscle. | | | | | | | | | |
| --- | --- | --- | --- | --- | --- | --- | --- | --- | --- |
| **Subject** | **EP** | **LQ** | **LH** | **LTA** | **LTS** | **RQ** | **RH** | **RTA** | **RTS** |
| SID1 | 0-3+ | 7.27 | 8.00 | 3.00 | 4.80 | 7.16 | 7.93 | 8.00 | 9.00 |
|  | 3-0+ | 4.08 | 4.11 | 5.00 | 4.56 | 3.07 | 3.38 | 4.69 | 6.36 |
| SID2 | 0-3+ | 1.61 | 1.01 | 0.41 | 0.89 | 2.14 | 2.25 | 2.30 | 2.01 |
|  | 3-0+ | 1.06 | 0.70 | 0.50 | 0.50 | 1.82 | 1.23 | 0.92 | 0.95 |
| SID3 | 0-3+ | 3.15 | 3.80 | 5.86 | 6.78 | 4.19 | 4.18 | 7.86 | 7.68 |
|  | 3-0+ | 1.71 | 1.08 | 1.78 | 3.28 | 3.74 | 2.83 | 2.74 | 3.43 |
| SID4 | 0-3+ | 6.48 | 6.07 | 8.00 | 7.00 | 5.56 | 5.39 | 7.00 | 6.00 |
|  | 3-0+ | 3.27 | 2.90 | 4.80 | 5.07 | 3.22 | 3.41 | 5.67 | 5.46 |
| SID5 | 0-3+ | 5.44 | 5.32 | 7.06 | 7.44 | 4.09 | 4.22 | 6.76 | 6.62 |
|  | 3-0+ | 8.36 | 4.37 | 2.96 | 3.75 | 5.89 | 3.40 | 2.28 | 2.12 |
| SID6 | 0-3+ | 8.03 | 7.88 | 7.00 | 2.50 | 8.26 | 7.65 | 9.20 | 9.40 |
|  | 3-0+ | 4.68 | 5.65 | 6.60 | 7.00 | 5.87 | 6.00 | 7.00 | 6.20 |
| SID7 | 0-3+ | 4.43 | 5.06 | 5.15 | 5.51 | 4.80 | 5.16 | 6.17 | 6.30 |
|  | 3-0+ | 2.06 | 2.12 | 3.51 | 3.48 | 3.37 | 1.99 | 3.41 | 3.19 |
| SID8 | 0-3+ | 7.08 | 8.56 | 10.00 | NA | 10.00 | 8.35 | 10.00 | 10.00 |
|  | 3-0+ | 5.85 | 3.56 | 5.02 | 6.39 | 5.93 | 2.18 | 4.50 | 6.70 |
| SID9 | 0-3+ | 10.00 | 10.00 | NA | NA | NA | 9.00 | 1.00 | NA |
|  | 3-0+ | 6.46 | 6.36 | 6.40 | 6.73 | 5.53 | 5.61 | 7.78 | 7.00 |
| SID10 | 0-3+ | 8.00 | 8.24 | 10.00 | 8.20 | 6.10 | 6.98 | 7.00 | 7.80 |
|  | 3-0+ | 4.28 | 3.84 | 4.23 | 4.20 | 2.15 | 2.31 | 3.56 | 3.29 |

| Supplementary Material 2. Summary of Monosynaptic Maximum Response [mVpp] for each subject, electrode position (EP) and muscle. NA indicates that no reflexes were elicited on that muscle. | | | | | | | | | |
| --- | --- | --- | --- | --- | --- | --- | --- | --- | --- |
| **Subject** | **EP** | **LQ** | **LH** | **LTA** | **LTS** | **RQ** | **RH** | **RTA** | **RTS** |
| SID1 | 0-3+ | 0.46 | 0.33 | 0.07 | 0.14 | 1.23 | 2.31 | 0.08 | 0.06 |
|  | 3-0+ | 3.80 | 2.08 | 0.10 | 0.16 | 3.28 | 2.98 | 0.57 | 1.25 |
| SID2 | 0-3+ | 2.32 | 1.81 | 2.21 | 5.00 | 1.02 | 4.80 | 3.52 | 3.37 |
|  | 3-0+ | 2.34 | 1.57 | 2.44 | 4.99 | 1.20 | 4.66 | 4.22 | 3.59 |
| SID3 | 0-3+ | 1.04 | 0.92 | 0.41 | 0.90 | 1.06 | 1.00 | 0.41 | 0.66 |
|  | 3-0+ | 0.54 | 0.81 | 0.61 | 1.41 | 0.65 | 0.84 | 0.74 | 1.54 |
| SID4 | 0-3+ | 1.67 | 1.50 | 0.18 | 0.86 | 3.26 | 2.99 | 0.23 | 0.57 |
|  | 3-0+ | 2.78 | 1.69 | 0.50 | 1.41 | 4.07 | 3.48 | 0.81 | 2.10 |
| SID5 | 0-3+ | 3.59 | 3.54 | 1.60 | 3.21 | 3.34 | 3.16 | 0.50 | 2.58 |
|  | 3-0+ | 3.13 | 4.41 | 1.70 | 3.30 | 3.88 | 3.47 | 0.61 | 4.08 |
| SID6 | 0-3+ | 1.03 | 0.32 | 0.04 | 0.03 | 1.68 | 0.39 | 0.18 | 0.08 |
|  | 3-0+ | 1.45 | 1.30 | 0.29 | 0.42 | 1.43 | 3.28 | 0.51 | 0.95 |
| SID7 | 0-3+ | 0.36 | 1.96 | 0.42 | 0.84 | 0.50 | 0.87 | 0.54 | 1.09 |
|  | 3-0+ | 0.32 | 2.16 | 0.82 | 1.44 | 0.45 | 0.87 | 1.51 | 1.28 |
| SID8 | 0-3+ | 0.08 | 0.30 | 0.06 | NA | 0.05 | 0.37 | 0.07 | 0.03 |
|  | 3-0+ | 0.95 | 1.23 | 0.44 | 1.78 | 0.82 | 0.95 | 0.88 | 1.45 |
| SID9 | 0-3+ | 0.05 | 0.03 | NA | NA | NA | 0.11 | 0.02 | NA |
|  | 3-0+ | 4.88 | 1.65 | 1.67 | 0.45 | 3.20 | 0.92 | 1.49 | 1.68 |
| SID10 | 0-3+ | 0.36 | 0.87 | 0.20 | 0.17 | 1.68 | 2.68 | 0.26 | 0.19 |
|  | 3-0+ | 1.41 | 3.73 | 0.34 | 0.34 | 2.18 | 4.12 | 0.71 | 0.40 |

| Supplementary Material 3. Summary of Polysynaptic Threshold [V] for each subject, electrode position (EP) and muscle. NA indicates that no reflexes were elicited on that muscle. | | | | | | | | | |
| --- | --- | --- | --- | --- | --- | --- | --- | --- | --- |
| **Subject** | **EP** | **LQ** | **LH** | **LTA** | **LTS** | **RQ** | **RH** | **RTA** | **RTS** |
| SID1 | 0-3+ | NA | NA | NA | NA | NA | NA | 10.00 | 9.00 |
|  | 3-0+ | NA | 7.00 | 5.00 | 3.00 | NA | 5.00 | 5.00 | 5.00 |
| SID2 | 0-3+ | NA | NA | 1.50 | 0.90 | NA | 0.50 | 0.70 | 1.80 |
|  | 3-0+ | 3.00 | 0.60 | 0.60 | 0.60 | 3.00 | 0.60 | 0.50 | 0.90 |
| SID3 | 0-3+ | NA | 7.00 | NA | NA | NA | 7.00 | 8.00 | 6.00 |
|  | 3-0+ | NA | 4.00 | 6.00 | 5.00 | 9.00 | 6.00 | 9.00 | 5.00 |
| SID4 | 0-3+ | NA | 8.00 | 7.00 | 6.00 | 7.00 | 6.00 | 7.00 | 7.00 |
|  | 3-0+ | 7.00 | 5.00 | 4.00 | 4.00 | 5.00 | 5.00 | 5.00 | 5.00 |
| SID5 | 0-3+ | 8.00 | 7.00 | 8.00 | 7.00 | NA | NA | NA | NA |
|  | 3-0+ | 10.00 | 8.00 | NA | 6.00 | NA | NA | NA | 7.00 |
| SID6 | 0-3+ | NA | NA | NA | NA | NA | NA | NA | 10.00 |
|  | 3-0+ | NA | 8.00 | NA | 10.00 | NA | 10.00 | 8.00 | 7.00 |
| SID7 | 0-3+ | NA | NA | NA | NA | NA | NA | 7.00 | 8.00 |
|  | 3-0+ | NA | 3.00 | 3.00 | 3.00 | NA | 3.00 | 5.00 | 3.00 |
| SID8 | 0-3+ | NA | NA | NA | NA | NA | NA | NA | NA |
|  | 3-0+ | 7.00 | 7.00 | 7.00 | 7.00 | 7.00 | 7.00 | 7.00 | 7.00 |
| SID9 | 0-3+ | NA | NA | NA | NA | NA | NA | NA | NA |
|  | 3-0+ | 7.00 | 8.00 | 7.00 | 7.00 | 7.00 | 7.00 | 8.00 | 6.00 |
| SID10 | 0-3+ | NA | 10.00 | 10.00 | 9.00 | 10.00 | 8.00 | 8.00 | 8.00 |
|  | 3-0+ | NA | 5.00 | 6.00 | 4.00 | 4.00 | 4.00 | 3.00 | 3.00 |

| Supplementary Material 4. Summary of Polysynaptic Maximum Response [µV·s] for each subject, electrode position (EP) and muscle. NA indicates that no reflexes were elicited on that muscle. | | | | | | | | | |
| --- | --- | --- | --- | --- | --- | --- | --- | --- | --- |
| **Subject** | **EP** | **LQ** | **LH** | **LTA** | **LTS** | **RQ** | **RH** | **RTA** | **RTS** |
| SID1 | 0-3+ | NA | NA | NA | NA | NA | NA | 0.49 | 2.95 |
|  | 3-0+ | NA | 0.84 | 2.13 | 3.62 | NA | 2.12 | 2.07 | 2.42 |
| SID2 | 0-3+ | NA | NA | 1.20 | 2.85 | NA | 1.37 | 1.08 | 3.04 |
|  | 3-0+ | 12.04 | 10.74 | 1.28 | 2.06 | 5.52 | 1.79 | 0.94 | 2.38 |
| SID3 | 0-3+ | NA | 5.27 | NA | NA | NA | 2.70 | 3.84 | 1.14 |
|  | 3-0+ | NA | 5.27 | 0.88 | 0.61 | 5.97 | 2.63 | 9.52 | 0.44 |
| SID4 | 0-3+ | NA | 3.18 | 3.89 | 6.05 | 3.03 | 3.76 | 3.03 | 4.56 |
|  | 3-0+ | 1.72 | 3.37 | 6.05 | 9.44 | 6.01 | 6.55 | 5.81 | 7.72 |
| SID5 | 0-3+ | 1.13 | 0.86 | 0.60 | 2.24 | NA | NA | NA | NA |
|  | 3-0+ | 0.51 | 0.48 | NA | 1.79 | NA | NA | NA | 1.70 |
| SID6 | 0-3+ | NA | NA | NA | NA | NA | NA | NA | 0.98 |
|  | 3-0+ | NA | 2.47 | NA | 0.45 | NA | 0.80 | 4.52 | 5.68 |
| SID7 | 0-3+ | NA | NA | NA | NA | NA | NA | 1.26 | 1.19 |
|  | 3-0+ | NA | 0.77 | 5.17 | 0.44 | NA | 2.99 | 1.08 | 1.56 |
| SID8 | 0-3+ | NA | NA | NA | NA | NA | NA | NA | NA |
|  | 3-0+ | 7.15 | 5.30 | 6.60 | 4.13 | 8.29 | 7.64 | 7.71 | 5.25 |
| SID9 | 0-3+ | NA | NA | NA | NA | NA | NA | NA | NA |
|  | 3-0+ | 2.01 | 2.64 | 3.72 | 7.08 | 1.86 | 0.62 | 0.97 | 10.71 |
| SID10 | 0-3+ | NA | 1.50 | 0.88 | 2.17 | 0.61 | 1.75 | 4.61 | 3.33 |
|  | 3-0+ | NA | 4.88 | 0.58 | 8.49 | 1.26 | 4.39 | 2.59 | 5.29 |
